# Rapid Biochemical Analysis of Postmortem Serum and Myocardial Homogenates—An Exploratory Study

**DOI:** 10.3390/biom15101483

**Published:** 2025-10-21

**Authors:** Niki Sarri, Henrik Druid, Ali-Reza Rezaie, Klaske Osinga, Nargis Sultana, Kanar Alkass

**Affiliations:** 1 Swedish National Board of Forensic Medicine, 17165 Solna, Sweden; niki.sarri@igp.uu.se (N.S.); ali.rezaie@rmv.se (A.-R.R.); kanar.alkass@ki.se (K.A.); 2 Department of Oncology-Pathology, Karolinska Institutet, Nobels v, 15A, 17177 Stockholm, Sweden; klaske.osinga@gmail.com; 3 Division of Clinical Chemistry and Pharmacology, Department of Biomedical and Clinical Sciences, Faculty of Medicine, Linköping University, 58185 Linköping, Sweden; nargis.sultana@regionostergotland.se

**Keywords:** biomarkers, sudden cardiac death, postmortem, tissue homogenate, clinical chemistry, stability, proteomics

## Abstract

Postmortem diagnosis of sudden cardiac death (SCD) may escape detection due to the absence of thrombi and slow development of structural and immunohistochemical changes. Therefore, this study explores the possibility of analyzing relevant clinical chemistry biomarkers in myocardial homogenates and serum. Following an initial pilot study, myocardial samples from 113 autopsy cases were homogenized with distilled water, T-PER or 2 M urea. Aspartate aminotransferase (AST), alanine aminotransferase (ALT), creatine kinase (CK-MB), lactate dehydrogenase (LDH), orosomucoid and total protein were analyzed with an IndikoPlus and a subset was also analyzed with a Roche Cobas 8000 c701 analyzer, which also provided results for cardiac Troponin T, myoglobin and NT-proBNP. Although the yields varied with different extraction buffers depending on the analyte, distilled water was often as effective as T-PER and 2 M urea extraction for most analytes. Biomarker levels were consistently higher in the myocardial homogenates than in serum. Proteomic profiling on a subset confirmed higher concentrations of the cardiac markers in the tissue samples than in serum. Finally, we investigated whether selected markers could support the diagnosis of acute cardiac disease by classifying cases as sudden cardiac death (SCD) or controls. There was no significant difference in serum concentrations of the selected biomarkers between SCD cases and controls, whereas a significant loss of several markers was observed in SCD myocardial samples as compared to controls. Hence, our results suggest that analysis of tissue homogenates is likely better for detecting early ischemia, and we show that an in-house benchtop multi-analyzer can provide rapid results to assist the pathologist’s decision-making during autopsy.

## 1. Introduction

**Figure 1:**
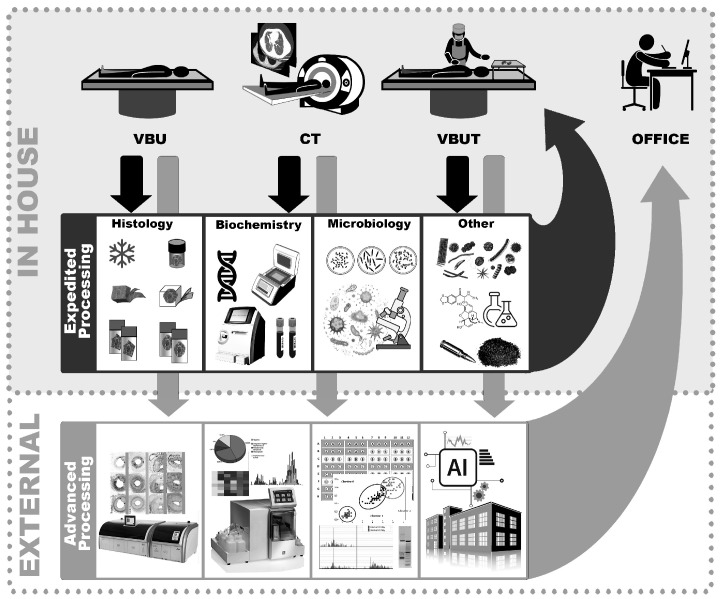
Proposed workflow for improved autopsy investigation; the upper panel represents analyses that can be performed in-house and provide rapid results. The lower panel includes analyses that may need to be performed at external laboratories. Abbreviations: V = vitreous, B = blood, U = urine, T = tissue, CT = computer tomography; AI = artificial intelligence.

An autopsy has long been considered the gold standard for identifying the cause of death [1]. However, traditional autopsy work still focuses on morphological changes, which may fail to identify certain acute medical conditions that follow a rapid course since structural changes require time to develop [2]. In contrast to structural alterations, many biochemical changes occur rapidly, either through translocation of proteins and other molecules within cells and tissues or by leakage from damaged tissue into the bloodstream [3]. Clinical chemistry is used extensively in primary care and hospitals to detect biochemical changes, but access to tissue samples from living subjects is limited; therefore, reference levels in serum/plasma or other accessible fluids are used as proxies. Postmortem biochemistry has been studied for decades but remains underutilized in practical casework [4] and published biomarker levels are exclusively from body fluids, mainly serum, pericardial fluid, cerebrospinal fluid, and vitreous fluid. In addition, rapid analysis with point-of-care instruments may aid decision-making during autopsy [5,6]. While various options exist, including spot tests and handheld analyzers, these often analyze only one marker at a time [7,8]. Our focus is on benchtop analysis to provide rapid results for multiple markers simultaneously. That said, there is a need for more analytical tools to aid postmortem diagnostics, and naturally not all need to provide instant results; rather, some have to be very accurate, particularly if the results may be presented as evidence in court. Figure 1 represents a schematic illustration of a strategy to improve future autopsy work. In this article we focus on rapid analysis that can provide leads to the pathologist regarding which investigative tracks may be important to follow and which ones are not supported by quick tests.

At both clinical and forensic pathology departments, many deaths are unwitnessed and recent medical information is often lacking. A proportion of these cases are suspected sudden cardiac deaths (SCD) [9]. SCD is defined as an unexpected natural death from a cardiac cause occurring within a short time, usually within one hour from symptom onset [10,11,12]. In some cases, acute myocardial ischemia (AMI) can be detected macro- or microscopically, but if the survival time is short, these changes may not be visible [13]. In other instances, an unwitnessed acute cardiac death due to arrhythmia may not be detectable at all, and acute heart failure may also escape detection by traditional autopsy methods [14].

Clinical chemistry diagnostic tests are generally developed for living subjects, and their performance on postmortem samples requires further investigation. A study on marker combinations recently reported an excellent prediction of acute myocardial ischemia by analyzing a combination of biomarkers [15], but these results have not yet been confirmed by other studies. Since all organs are accessible at autopsy, early-phase changes are more likely to be detected by analyzing the affected tissue directly. We investigated the performance of a benchtop instrument, Indiko™ Plus (Thermo Fisher Scientific, Vantaa, Finland), on cases of SCD and deaths from other known causes. Due to its analytical principles, not all desired cardiac ischemia markers can be analyzed with this instrument, such as cTnT and myoglobin. We selected AST, ALT, CK-MB, and LDH, all of which will show changes upon cardiac ischemia. Using a different instrument, cTnT, myoglobin, and NT-proBNP were also analyzed in a subset of cases. The main objectives of this study were to determine: (a) whether the Indiko instrument can provide reliable results from postmortem serum and myocardial homogenates; (b) whether the degree of arteriosclerosis in the supporting artery is related to biomarker concentrations in the corresponding myocardial samples; (c) whether postmortem interval and cardiac massage affect biomarker levels; and (d) whether freeze-thawing of tissue causes changes in biomarker levels. Most importantly, we investigated whether certain preanalytical treatments could increase biomarker yields from myocardial homogenates and whether removal of hemoglobin from serum could improve analytical results in hemolyzed samples. Additionally, we aimed to compare the performance of the Indiko instrument with a standard clinical chemistry multi-analyzer, and to assess whether the selected biomarkers could discriminate SCD cases from those with other causes of death.

## 2. Materials and Methods

### 2.1. Study Design and Study Population

Two prospective, explorative studies were conducted from spring 2023 through spring 2024. First, a pilot study was performed on 43 autopsy subendocardial samples collected at the Department of Forensic Medicine in Stockholm, comprising 35 males and 9 females with an average age of 57.0 years (Table 1).

The main study was based on a separate cohort of 113 autopsy cases, from which subendocardial samples from the mid posterior wall of the left ventricle were collected at the same department, comprising 83 males and 30 females; average age 56.7 years (Table 2). In 91 of these (64 males and 27 females), a sample from the mid anterior wall was also collected.

It should be noted that both cohorts included cases with a variety of causes of death and several of them with additional medical conditions that in most cases were not considered to have contributed to death. This means we used an unselected sample for the studies of different preanalytical treatments to increase the likelihood that the results would be as generally applicable as possible to a typical forensic or clinical pathology autopsy population. Inclusion criteria for evaluating different extraction conditions were based solely on body freshness, i.e., absence of decomposition (although limited greenish discoloration of the skin on the lower abdomen was accepted). Hence, the main purpose of this study was to explore the possibility of analyzing available clinical chemistry markers in tissue homogenates, i.e., where a particular pathology may be present. Since we focused on myocardium, we selected cardiac markers that have been used for decades, although developed for blood (plasma or serum) samples, to determine whether these could be analyzed with a benchtop clinical chemistry multi-analyzer.

To further investigate if any of these markers may show a change in the myocardium before a rise in serum, we classified cases as SCD or control (or not included in any of these groups). Inclusion criteria for SCD were a cardiac cause of death determined by the forensic pathologist and circumstances supporting a cardiac death. Circumstances supporting SCD include cases where the subject had reported chest pain and patients with a known heart condition found dead after physical exertion. Hence, we did not classify cases as SCD based solely on a cardiac cause of death or solely on circumstances suggesting a sudden cardiac event. Equally important for the assessment was also the exclusion of other competing alternative causes of death. Autopsy reports, including microscopic examination, toxicological analytical results, police reports, and when available medical records, were perused. In a majority of cases, we retrieved and reviewed medical records, but most cases had no very recent visits. Probable SCD cases with competing causes of death were excluded. Cases were classified as controls when cause of death was unrelated to SCD; exclusion criteria were age <18 years, suspected homicides, severe decomposition, significant liver steatosis, liver cirrhosis, or chest trauma. Some cases could not be reliably classified, e.g., due to insufficiently detailed description in the autopsy protocol and/or (more often) unclear circumstances. This is why some cases in Table 1 and Table 2 with a cardiac death diagnosis, such as AMI, coronary arteriosclerosis, or enlarged heart were classified as SCD, whereas other cases with the same diagnosis were not included. Basically, we followed the SCD classification by Basso et al. [16], although two cases of pulmonary embolism (M06, M40) were included in the control group, since both had saddle emboli that most likely had caused a very rapid death with no chance to cause biochemical changes in the myocardium. We also included five cases (M38, M58, M62, M63, and M77) in the control group despite the presence of heart pathologies, since their deaths from other causes were rapid.

### 2.2. Postmortem Interval Estimation (PMI)

Police reports and medical records, when available, were perused to retrieve information about time found dead, last seen alive, or time of witnessed death. Most deaths occurred indoors, and a temperature of 22 °C was assumed, based on temperatures recorded at many previous scene investigations. All bodies were then stored in cold rooms at the forensic medicine department at a temperature of 6 °C before autopsy. A simple equation was used to generate temperature-corrected Postmortem Interval (tcPMI), which should reflect the time between death and sampling if the temperature had been constant at 22 °C: tcPMI = warm time + (6/22 × cold time). In a proportion of cases, the exact time of death was known. If death was unwitnessed, we accepted a time of death occurring during a time interval that was <10% of the warm time (until the body was placed in cold storage), and the midpoint of this interval was used as the estimated time of death.

### 2.3. Sample Collection and Preparation

**Figure 2:**
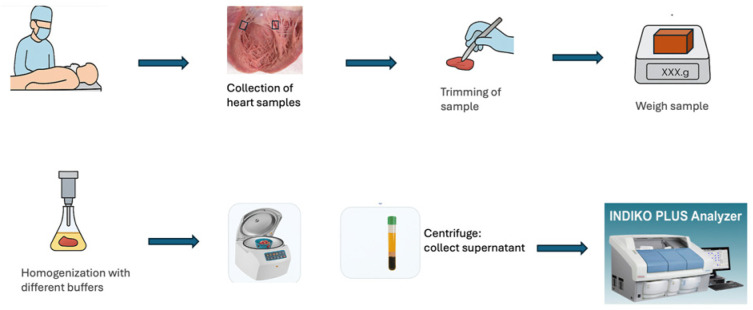
A schematic illustration of the workflow. At autopsy, blood and myocardial samples from the posterior wall were consistently collected. In 91 cases from the main study, an additional sample from the anterior wall was also collected. Myocardial samples were homogenized with UltraTurrax with different buffers and subsequently centrifuged. The supernatant (diluted 1:20 in the main study) was analyzed using the Indiko multi-analyzer.

Figure 2 provides a summary of the workflow. All myocardial samples were collected approximately 0–6 mm from the endocardium, approximately 3 cm below the valves. From all cases, a femoral vein blood sample (for serum preparation) was also collected. In the pilot study, 1 g of myocardium was homogenized with 2 mL of the following solvents: distilled water (dH_2_O), PBS, 8 M urea in PBS, 0.1% tween in PBS, 8 M urea, and 20% SDS, using ULTRA-TURRAX homogenizer VDI 12 (IKA, Oxford, UK). Next, the homogenates were centrifuged at 3000× *g* for 20 min at 4 °C, and the supernatant was analyzed. To assess the effect of freeze-thawing on postmortem protein stability, one portion of 12 tissue samples was homogenized directly, and another portion was frozen for one week at −20 °C before homogenization. To this end, we chose to analyze creatinine and total protein, the former being a small molecule expected to remain stable, and the latter as a general marker of protein integrity. Pronounced hemolysis is quite common in serum from postmortem blood. To assess possible interference of hemoglobin in the serum with analysis, 23 femoral blood aliquots underwent hemoglobin removal with HemogloBind™ (Gentaur, 337-HO145-05, BSG, Monmouth Junction, NJ, USA). Briefly, equal parts of HemogloBind™ suspension and femoral blood were mixed, incubated for 10 min, and centrifuged at 3000× *g* for 2 min. For comparison, the same blood samples were mixed with dH_2_O and treated similarly.

In the main study, the myocardial homogenates were prepared at a 1:20 ratio (200 mg tissue: 4 mL buffer) in dH_2_O, T-PER (a proprietary detergent in 25 mM bicine, 150 mM NaCl; pH 7.6, Thermo Fisher Scientific, Vantaa, Finland, #78510), and Tris-Urea (25 mM Tris-HCl), or 2 M Urea (Thermo Fisher Scientific, #424585000; pH 7.4) buffers supplemented with HALT protease inhibitor cocktail (Thermo Fisher Scientific, #78438). T-PER is a mild detergent that maximizes protein solubilization without compromising enzymatic activity. After homogenization with ULTRA-TURRAX homogenizer VDI 12 (VWR), and centrifugation at 3000× *g* for 20 min at 4 °C, the supernatants were collected and stored at −20 °C. Serum was prepared by centrifugation of femoral vein blood at 3000× *g* for 20 min and stored at −20 °C.

### 2.4. Laboratory Assays

The biomarkers CK-MB, AST, total protein, creatinine, haptoglobin, fructose, and orosomucoid (alpha 1-acid glycoprotein) were analyzed in myocardial homogenates and serum in the pilot study using an Indiko™ (Thermo Fisher Scientific) instrument. In the main study, CK-MB, AST, ALT, LDH (all of which typically show temporal changes in serum concentration upon a significant myocardial injury), orosomucoid and total protein levels in tissue homogenates and serum samples were analyzed using an Indiko™ Plus (Thermo Fisher Scientific) instrument. The analytical principle for CK-MB, AST, ALT, and LDH is based on enzymatic reactions in which their specific substrates are converted through serial reactions that finally generate NADH/NADPH, and the spectrophotometrically measured absorbance at 340 nm is directly proportional to the enzyme activity in the samples. For determining orosomucoid concentration, Indiko utilizes a turbidimetric assay. To determine total protein concentration, the Indiko instrument uses a colorimetric method, in which the colored protein product is measured at 540 nm. The rationale for including creatinine, haptoglobin, and fructose (in the pilot study), and orosomucoid and total protein was that these are expected to be unaffected by an acute cardiac event, but also to determine whether different extraction alternatives affected the yields of the small molecules creatinine and fructose.

Measurements of a subgroup of sixteen samples were later independently analyzed at the Clinical Chemistry laboratory at Linköping University Hospital, Sweden, using a Roche Cobas 8000 c701 analyzer (Roche Diagnostics, West Sussex, UK). With this instrument, concentrations of cardiac cTnT, NT-proBNP, and myoglobin in tissue homogenates and serum were also determined, as these are also used for detecting cardiac conditions. The levels of CK-MB, cTnT, NT-proBNP, and myoglobin were detected using electrochemiluminescence immunoassay (ECLIA), which employs a sandwich immunoassay technique. The analysis of AST, ALT, LDH, and orosomucoid performed with the Roche Cobas 8000 c701 analyzer followed the same principles as those used with the Indiko instrument.

### 2.5. Mass Spectrometry Analysis (Proteomics)

Ten cardiac tissue homogenates and ten corresponding serum samples were analyzed in a buffer containing 10% β-octyl glucopyranoside. Serum samples were treated with a perchloric acid depletion protocol. For both sample types, proteins were reduced, alkylated, and subjected to on-filter digestion with trypsin using 3kDa centrifugal spin filters (Merck Millipore, Dublin, Ireland). Peptides were collected, dried using a Speedvac system, and reconstituted in 0.1% formic acid. Peptides were separated on a C18 reverse-phase column using a 150-min gradient. Analysis was performed on a QEx-Orbitrap mass spectrometer (Thermo Finnigan, San Jose, CA, USA) using HCD for tandem mass spectrometry [17]. Raw data were analyzed using MaxQuant 1.5.1.2 software. Proteins were identified using the Homo sapiens proteome from Uniprot [18].

### 2.6. Statistical Analysis

Due to the small sample sizes and the non-normal distribution of the data, non-parametric tests were used. For the freeze/thaw and Hemoglobind™ incubation serum experiments, differences between groups were assessed using the Wilcoxon signed-rank test. For the pretreatment optimization experiment, group differences were initially screened using the Kruskal–Wallis test. When a significant difference was found, this was followed by pairwise Wilcoxon rank-sum tests with Bonferroni–Holm correction for multiple comparisons. All tests were two-sided, and a *p*-value < 0.05 was considered statistically significant unless otherwise specified. Statistical analyses were conducted using R version 4.3.1. For the comparison between the SCD group and the control group, the Mann–Whitney U test was used with Bonferroni-Holm correction. With this test the two groups were compared for every measured analyte. For the comparisons regarding degree of arteriosclerosis and biomarker levels in the corresponding homogenates, the Wilcoxon signed-rank test was used. Comparisons between laboratories were analyzed with both Pearson and Spearman rank correlation tests for linear and non-linear correlations.

### 2.7. Ethical Considerations

All subjects were deceased, and therefore informed consent could not be obtained. In Sweden the Autopsy Law (1995:832) states that tissue samples may be procured by the forensic pathologist if the analytical results may be of importance for the purpose of the autopsy or for method development. This investigation was conducted in compliance with the abovementioned law and conformed to the principles of the Declaration of Helsinki. The study was approved by the Swedish Ethical Review Authority (No 2018/101-31/1 and 2023-03463-01). These approvals imply that the ethical review boards accepted the procedures described in the applications, where it is stated that the samples are not anonymized, but as soon as all necessary information about the deceased subjects has been retrieved, cases are assigned a code; hence, no person-identifiable results are generated during further processing and compilation of data.

## 3. Results

### 3.1. Pilot Study

#### 3.1.1. HemogloBind Treatment

Creatinine levels remained largely unchanged between HemogloBind™-treated and dH_2_O-treated samples. Total protein levels decreased significantly after HemogloBind™ treatment (*p* < 0.001), which is explained by hemoglobin removal. Since simple dilution of samples with pronounced hemolysis with dH_2_O was as effective as HemogloBind™ treatment for obtaining analytical results, we decided not to use HemogloBind in these studies.

#### 3.1.2. Impact of Freeze–Thaw Cycles on Postmortem Stability of Proteins

**Figure 3:**
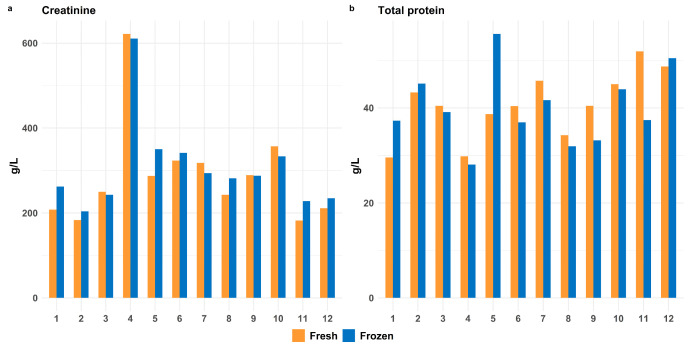
Effect of freezing and thawing on heart tissue levels of the selected biomarkers. (**a**) creatinine and (**b**) total protein. The *x*-axis shows case numbers, and the *y*-axis displays the measured values. Fresh tissue (orange) and tissue frozen at −20 °C for one week (blue) showed no significant differences (Wilcoxon Signed-Rank Test).

We compared biomarker levels in fresh tissue homogenates and samples stored for one week at −20 °C (Figure 3).

The Wilcoxon Signed-Rank Test showed no significant difference between fresh and frozen samples. Based on these findings, we decided to freeze samples before analysis to allow more samples to be analyzed in the same run.

#### 3.1.3. Efficacy of Different Myocardial Tissue Homogenization Conditions

**Figure 4:**
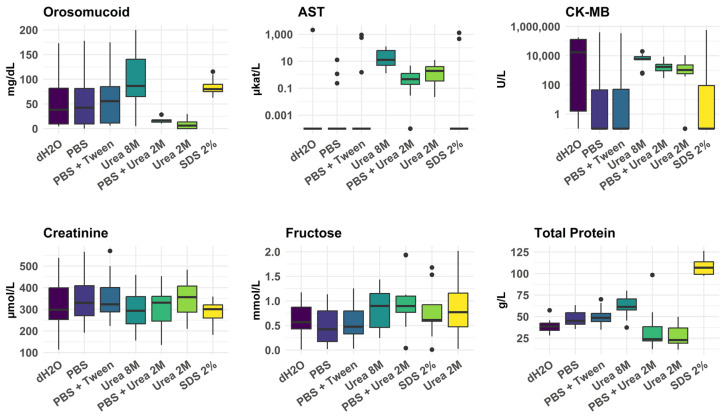
Effects of different homogenization treatments on the concentrations of the selected biomarkers in the pilot study. Results are presented for orosomucoid, AST, CK-MB, creatinine, fructose, and total protein. All 43 cases were analyzed. However, with the 8 M urea treatment, the Indiko instrument failed to report a result for many samples due to excessive viscosity of the extract.

In the pilot study, we assessed the impact of different homogenization treatments (Figure 4). None of the preanalytical treatments was consistently most efficient. Although 8 M urea often gave a good yield, it also frequently produced a sample with high viscosity, preventing analysis.

### 3.2. Main Study

#### 3.2.1. Evaluation of Extraction Methods for Effective Biomarker Yield from the Myocardium

**Figure 5:**
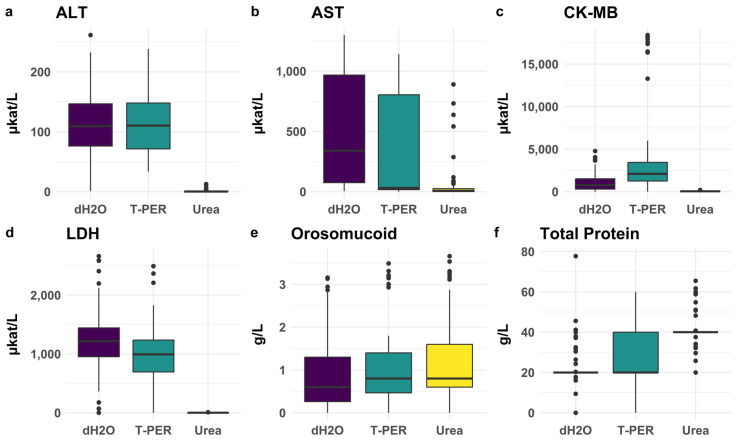
Impact of different homogenization treatments on the concentrations of the selected biomarkers (posterior wall), (**a**) ALT, (**b**) AST; (**c**) CK-MB, (**d**) LDH; (**e**) orosomucoid; (**f**) total protein. All 113 cases in the main cohort were included regardless of classification as SCD or controls (or neither). ALT activity showed no significant difference between dH_2_O and T-PER treatments (*p* = 0.954). dH_2_O yielded the highest median activity for AST and LDH (*p* = 0.013 and *p* < 0.0001, respectively), while T-PER produced the highest CK-MB activity (*p* < 0.0001). Significantly lower enzyme levels were obtained with 2 M urea compared to dH_2_O and T-PER (*p* < 0.0001).

To further optimize the extraction conditions for the posterior wall myocardial samples, we examined the effectiveness of three extraction solvents: dH_2_O, T-PER, and urea 2 M, see Figure 5.

dH_2_O produced results similar to T-PER for most analytes, whereas urea often resulted in poor yields. All pairwise comparisons between serum and the preanalytical treatments for ALT, LDH, and CK-MB in homogenates showed *p*-values < 0.0001, with serum levels being consistently lower. The extraction efficiency of heart biomarkers in the anterior wall mirrored the efficiency observed in the posterior wall (see below).

#### 3.2.2. Evaluation of Extraction Methods for Myoglobin, NT-proBNP and cTnT

**Figure 6:**
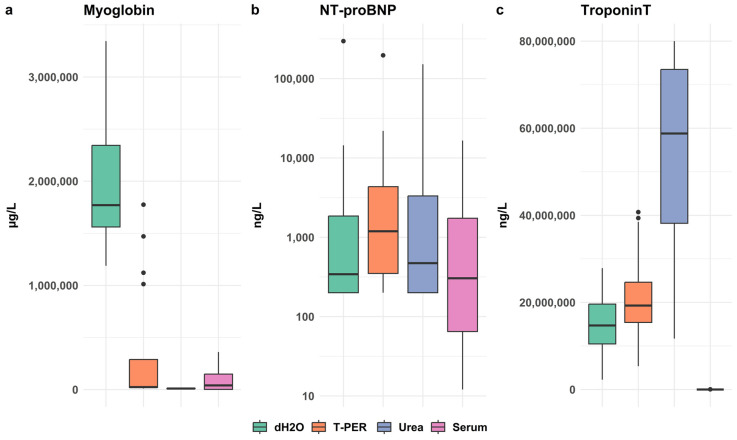
Biomarker levels, (**a**) myoglobin; (**b**) NT-proBNP; (**c**) troponinT, in myocardial tissue extracts from all cases in the main study were analyzed using the Cobas 8000 c701 platform. dH_2_O yielded the highest myoglobin levels (*p* < 0.001 vs. other conditions) but with a broad interquartile range. NT-proBNP levels were slightly higher with T-PER, although the differences were not statistically significant (*p* > 0.05). Extraction with 2 M urea resulted in markedly higher cardiac troponin T (cTnT) concentrations compared with dH_2_O and T-PER homogenates (*p* < 0.001), whereas dH_2_O and T-PER did not differ significantly (*p* = 0.056).

These biomarkers could not be analyzed with the Indiko instrument but were analyzed with the Cobas instrument at the Clinical Chemistry Laboratory in Linkoping, Sweden. The optimal extraction conditions varied for myoglobin, NT-proBNP, and cTnT, as shown in Figure 6.

#### 3.2.3. Comparative Biochemical Analysis of Posterior and Anterior Wall

**Figure 7:**
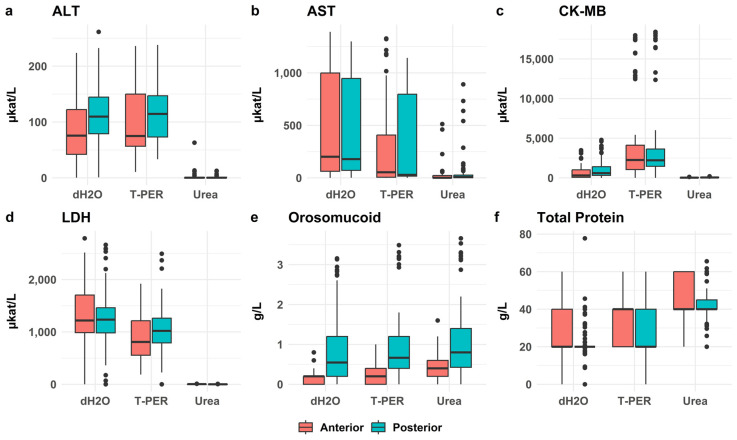
Concentrations of the selected biomarkers, (**a**) ALT, (**b**) AST, (**c**) CK-MB, (**d**) LDH, (**e**) orosomucoid, (**f**) total protein, in subendocardial samples from the anterior and posterior left ventricular walls. Using dH_2_O extraction, significantly lower concentrations were observed in anterior wall samples for ALT and CK-MB (*p* < 0.001). With T-PER, total protein levels were also significantly lower in the anterior wall compared with the posterior wall (*p* = 0.019). Using urea extraction, AST, CK-MB, and LDH showed significantly lower concentrations in the anterior wall (*p* < 0.005). For all three pretreatments, orosomucoid levels were consistently lower in the anterior wall than in the posterior wall (*p* < 0.001).

When examining results for all 113 cases, the anterior wall displayed almost consistently lower values than the posterior wall for most pretreatments. Although some differences were statistically significant, they were generally small (Figure 7).

#### 3.2.4. Cross-Laboratory Validation of Extraction Methods

**Figure 8:**
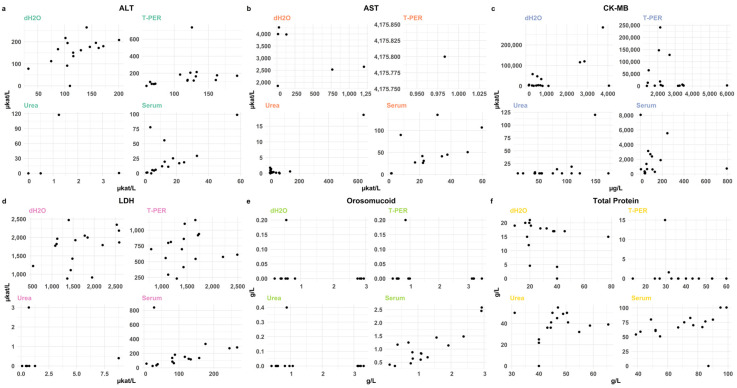
Comparison of selected biomarker levels of (**a**) ALT, (**b**) AST, (**c**) CK-MB, (**d**) LDH, (**e**) orosomucoid, (**f**) total protein, between the Indiko Plus and Cobas 8000 c701 analyzers. A total of 64 samples were analyzed. For dH_2_O-treated samples, median enzyme levels of ALT (*p* = 0.011), AST (*p* < 0.001), CK-MB (*p* < 0.001), and LDH (*p* < 0.001) were significantly higher with the Cobas instrument. Other treatments (T-PER and urea) and serum samples showed either similar or non-significant differences between instruments. Regression analysis demonstrated variable results for the homogenates, whereas most serum measurements showed fair correlations between the two systems.

The results are shown in Figure 8. The Cobas instrument reported significantly higher median levels of ALT, AST, and CK-MB than the Indiko instrument for dH_2_O homogenates. The T-PER- and urea-treated homogenates showed no statistically significant differences. We then performed a regression analysis to examine whether the biomarker levels measured in two independent laboratories (Stockholm, Linköping) agreed, see Figure 8. The best correlations were observed for 2 M urea extractions of ALT (Spearman 0.803), but several correlations were low. Overall, positive correlations were observed for ALT across all treatments, and for AST, CK-MB, and LDH, under certain extraction conditions.

#### 3.2.5. Comparison of Biomarkers Between SCD Cases and Controls

Comparisons between SCD cases and controls in posterior wall samples are shown in Table 3.

Cardiac biomarkers in posterior wall homogenates were generally lower in the SCD group compared to controls, except for CK-MB in T-PER extracts. With dH_2_O and urea extraction, CK-MB concentrations were significantly lower in SCD cases than in controls, and with dH_2_O and T-PER, LDH concentrations were also lower than in controls (*p* < 0.05 for these comparisons). In the anterior wall, AST, CK-MB, and LDH concentrations in SCD cases were significantly lower than in controls (*p* < 0.05) (see Table 3).

#### 3.2.6. Impact of CPR on Analytical Results

**Figure 9:**
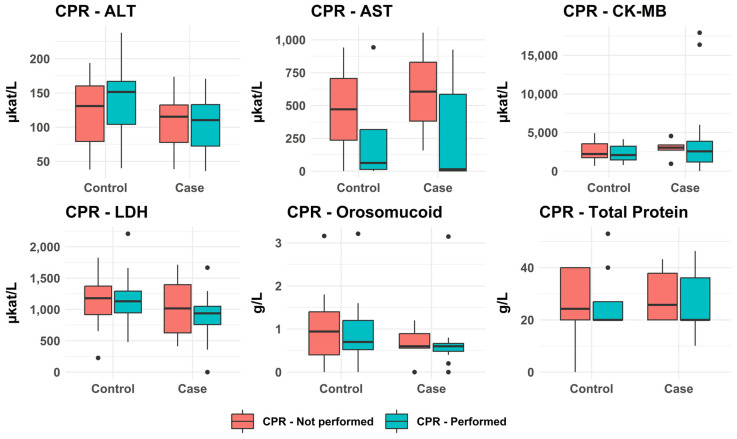
Effect of CPR on the concentrations of the selected analytes in the posterior myocardial homogenates. There were no significant differences in biomarkers between subjects who received CPR and those who did not.

As shown in Figure 9, median concentrations of all markers were generally lower in subjects who received CPR compared to those who did not receive CPR. However, the differences were usually small, and no differences reached statistical significance.

#### 3.2.7. PMI—tcPMI—Postmortem Interval Correlation with Biomarkers Levels

**Figure 10:**
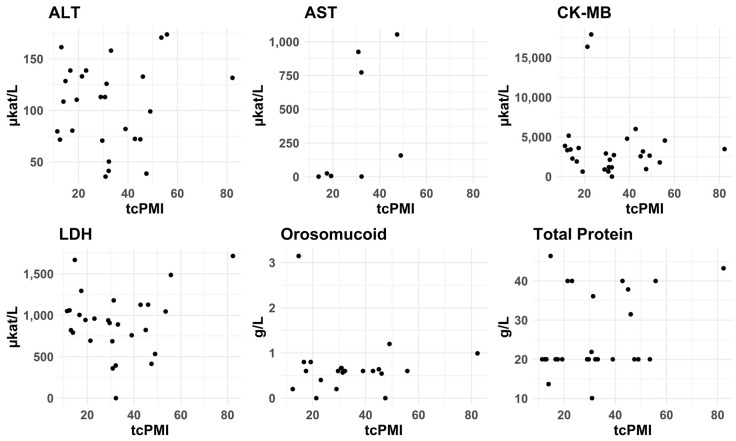
Effects of temperature-corrected postmortem interval (tcPMI) on concentration of the selected biomarkers in SCD cases. We did not observe any significant correlation between these measures of postmortem time and biomarker concentrations.

**Figure 11:**
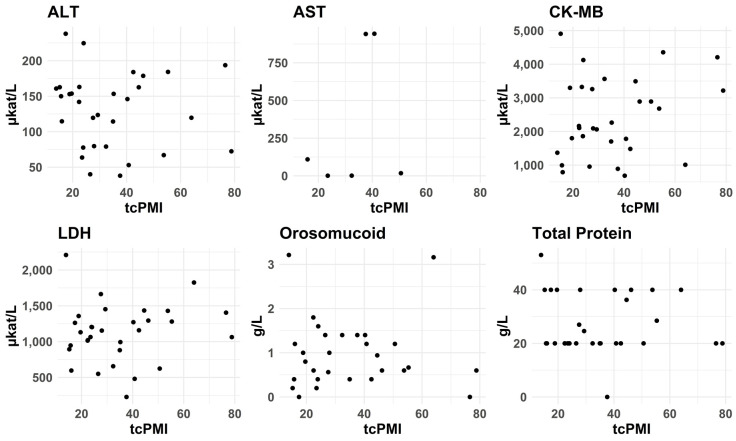
Effects of temperature-corrected postmortem interval (tcPMI) on concentration of the selected biomarkers in control cases. We conducted the same Pearson and Spearman correlation analyses as in Figure 10. We did not observe any significant correlation between these measures of postmortem time and biomarker concentration in this group either.

We conducted Pearson and Spearman correlation analyses to assess the possible linear and non-linear relationships between analyte values and postmortem crude interval (PMI), cold time (CT), and warm time (WT) for case and control groups from the left posterior wall, as well as temperature-corrected PMI (ctPMI). There were no significant correlations between the levels of the different biomarkers and any of these measures. For ctPMI, see Figure 10 and Figure 11. It should be noted, though, that we did not include decomposed cases.

#### 3.2.8. Comparison of Coronary Arteriosclerosis and Biomarker Levels

We compared the degree of arteriosclerosis of the left anterior descending coronary artery (LAD) and the right coronary artery with concentrations of the selected biomarkers in the anterior and posterior wall samples, respectively, but we did not find significant changes in the biomarker levels in the corresponding homogenates (Table 4).

#### 3.2.9. Proteomics

We performed proteomics analysis of 10 randomly selected cases to confirm the presence and concentrations of the cardiac biomarkers in myocardial samples and serum. In the cardiac tissue homogenates, a total of 1355 proteins were identified across all 10 samples. The number of identified proteins per sample ranged from 256 to 864, with an average of 589 proteins per sample. In the serum samples, a total of 1062 proteins were identified across all 10 samples. The number of identified proteins per sample ranged from 338 to 608, with an average of 434 proteins per sample. The concentrations of all analytes were much higher in the homogenates than in serum.

In fact, serum cTnT, CK-MB and AST were under the limit of quantitation, and only orosomucoid and myoglobin were consistently detected in serum from all cases, see Table 5.

## 4. Discussion

The Indiko instrument successfully delivered results for all selected biomarkers in most cases, both for serum samples and myocardial tissue homogenates. Homogenization, centrifugation and analysis could be completed within one hour, implying the possibility of implementing analysis of samples collected at autopsy and obtaining results before the examination of the body is completed. Furthermore, hemolysis of serum did not pose any issues, and freeze-thawing had no significant effect on concentrations of the examined biomarkers, which is in accordance with previous studies [19,20]. Cardiopulmonary resuscitation (CPR) did not affect biomarker levels, which aligns with previous studies [21,22,23]. Additionally, we did not find a correlation between postmortem interval and biomarker concentrations, which is also is consistent with results from other studies [22,24]. However, we excluded cases with significant decomposition, since there is an obvious risk for marker degradation, or at least a loss of enzymatic activity for those markers that are enzymes and are detected by their ability to catalyze reactions in the instrument. Hence, we do not know how well these markers will perform on decomposed cases. This will be an important objective for future studies.

The choice of method for extracting proteins and other components from a tissue sample is dependent on which molecules one wishes to obtain for analysis. To this end, different methods provide variable yields dependent on the characteristics of the target molecules, e.g., their size, physicochemical properties, and attachment to various cellular structures. In this context, the pioneering work by Fred S Apple and co-workers must be acknowledged. In the paper by Voss et al. [25], they showed that cTnT, CK-MB, and myoglobin concentrations in 14 regions of the healthy human heart were similar and that CK-MB and myoglobin were 99% cytosolic whereas 92% of troponin T was myofibril-bound. They further reported that the increase in troponins in serum paralleled a decrease in troponins in ischemic areas of the myocardium after coronary artery occlusion in dogs [26]. Another aspect to consider is that pretreatment may interfere with the function of the marker molecules. Several of the markers used in this study are enzymes, and the analytical methods of these depend on a preserved enzymatic activity of the protein. An additional aspect is the solubility of the marker in the extract. For instance, urea has been used extensively because it interacts both with polar and nonpolar residues, thereby stabilizing the solvation of the unfolded protein state [27], but excessively high urea concentration caused a high viscosity of the sample that prevented analysis in the pilot study. T-PER is a standard buffer for protein extraction from tissue samples [28]. When comparing pretreatment methods, extraction with dH_2_O was usually as effective as other extractions for most analytes. IndikoPlus generally reported higher concentrations of CK-MB extracted with dH_2_O than the Cobas instrument, but we have not been able to identify the explanation for this, since the analytical principle is the same in both instruments, although the calibration protocols are somewhat different. It is also possible that the instruments may have different sensitivities to matrix effects, given that both are designed to analyze serum rather than tissue homogenates. However, it should be kept in mind that this is an exploratory study, and repeated analysis on larger samples might not confirm this difference.

Urea generally provided the lowest yields, although it was most efficient for cTnT, whereas dH_2_O gave the best yields for myoglobin. This means that it is important for further studies to evaluate extraction alternatives for each new analyte. Such studies may include combinations of extraction agents to improve the yields; for instance, Frostadottir et al. [29] reported that addition of 8 M urea to a RIPA buffer increased protein yields from fresh frozen human peripheral nerve samples. Dilution of samples before analysis is a classical measure that often is automatically activated in standard clinical chemistry multi-analyzers, but since biomarker levels in myocardial homogenates differ from those in serum, testing different dilutions may be necessary to match the calibration range of the instrument’s analytical methods. Serum samples provided reliable results for nearly all analytes, with only rare failures. In contrast, heart homogenates showed occasional failures across all tested pretreatment methods, particularly in the pilot study.

Results for different analytes differed between analysis with Indiko and Cobas. One reason for the difference is that the Indiko results were obtained from analysis of the fresh samples, whereas the Cobas results were obtained from one of several aliquots of extracts that had been frozen for several months, and which might not have been perfectly mixed before or after freezing.

Sacco et al. [24] and Kutlu et al. [30] recently reported that a combination of cardiac injury serum markers could be used and demonstrated high sensitivity and specificity. However, confirmatory studies are needed, since several previous studies have not shown such good separation of cases and controls. Most likely, case selection is the critical factor, i.e., that SCD cases with certainty had extensive area(s) of cardiomyocyte necrosis and that controls had perfectly healthy hearts. In the study by Kutlu et al. [30], the control group subjects were significantly younger and had significantly lower heart weights, whereas in our study, the groups were much more similar in these and other respects, which we believe is more relevant in practical casework if one wishes to apply biochemistry as a tool to distinguish cases with similar characteristics. Furthermore, in addition to a loss of cardiac biomarkers from injured cardiomyocytes, it has also been observed that there are dynamic changes in levels over time, most likely due to de-novo production by cells in the penumbra zone; this temporal variation was already reported in experiments by Sharkey in 1991 [31]. The lower amounts of several analytes in the anterior left ventricular wall sample compared to the posterior wall sample may be explained by postmortem redistribution when most victims are placed in a supine position [26,27,28,32,33,34], implying sedimentation of blood and lymph possibly rich in leaked cardiomyocyte contents.

Today, repeated measurements of cTnT are used extensively in hospitals upon suspicion of myocardial ischemia. Even if there are sensitive immunoassays for troponin analysis, the most reliable method for analysis of cTnT is by electrochemiluminescence, which the Indiko instrument cannot offer. We therefore decided to analyze AST, ALT, CK-MB, and LDH. The pattern of these biomarkers in serum was used extensively in the past to confirm/assess AMI [35,36]. The advantage of analyzing the biomarkers also in cardiac homogenates is that it is likely that the decrease in concentrations in the myocardium will precede the increase in serum levels, since losses from the necrotic cardiomyocytes first will enter the local blood and lymph vessels in the heart before reaching the large volume of blood in the circulation.

Following an acute ischemic heart event, serum levels of CK-MB and cTnT will not increase immediately [37,38,39]; hence, there is a potential to discover acute myocardial ischemia by measuring the loss of biomarkers in myocardial homogenates. For AST, ALT and LDH, their rise in serum is even more delayed. This delay may explain why we did not find increased serum levels of these markers if many of the SCD cases died early after symptom onset (Table 3).

Whereas we failed to find increased serum levels of the selected biomarkers, in the tissue homogenates, CK-MB and LDH, and even ALT levels were significantly lower in SCD cases than in controls (Table 3), which lends support for analysis of biomarkers in myocardial homogenates.

Several factors limited the classification accuracy, including (a) lack of precise information of ischemic myocardial area; (b) arrhythmia as the cause of death; and (c) non-cardiac causes of death where the circumstances surrounding death mimicked SCD. The inclusion of controls also provided some challenges, but we selected cases where there was an obvious non-cardiac-related cause of death and where death was either immediate or followed a rapid course. As pointed out in Section 2, five of the controls did show heart pathology (enlarged heart or arteriosclerosis), which may be considered a limitation. However, all these deaths had another explanation, and we consider it very unlikely that their pre-existent heart pathologies would coincidentally have resulted in a myocardial injury shortly prior to death. Another limitation is that genetic testing had not been requested by the responsible pathologist in these cases. Although such testing will not prove that a functional deficit caused death, we acknowledge that this type of information is important, just like other forms of medical history that can imply a risk for sudden cardiac death. Another drawback was that we collected samples after the pathologist had collected samples for the death investigation; hence, some of our samples were obviously not taken from the center of visibly suspected myocardial areas. Furthermore, even if we performed proteomics analysis on a subset of cases, the purpose was not to compare SCD and controls but to determine whether we could confirm higher concentrations of the investigated cardiac markers in the myocardium than in serum. Indeed, we observed that biomarker levels, as measured with this quantitative mass spectrometry, were consistently higher in the myocardial homogenates than in serum, which aligns with the protein expression data reported in the Human Protein Atlas (www.proteinatlas.org) [40]. We believe that data from existing and additional proteomics analyses of myocardial homogenates can be used to identify more suitable markers of ischemia and inflammation for analysis in myocardial homogenates than the currently used cardiac injury markers, which are all intended for serum analysis. In such efforts, it will be important to consider the size, shape and localization of candidate molecules, e.g., whether they are bound to myofibrils, integrated in the cell membrane or are cytosolic. These and other factors, such as the myocard:serum concentration ratio, will impact how easily molecules leak into the blood. Further, proteomics analysis may also identify metabolites that are formed in small amounts and therefore difficult to analyze in serum, and there may be reactive products in surviving cells associated with ischemic injury that are not lost to any significant extent to the circulation, but which might be suitable targets in myocardial extracts.

In summary, we show that clinical chemistry cardiac markers can be analyzed in myocardial homogenates and that, with a benchtop instrument, results can be obtained in less than an hour, thus assisting the pathologist in the decision-making regarding which further investigative tracks to follow before the autopsy is completed.

## 5. Conclusions

Analysis of cardiac markers in myocardial samples collected at autopsy is seemingly a feasible approach to detect early myocardial ischemia, and possibly also acute inflammation. To this end, a systematic search for, and testing of, promising target molecules present in the myocardium, is warranted. Once suitable target molecules are identified, it will be important to examine the possible need for pre-treatment, since concentrations, both pathological and normal, in the extract into the instrument must match the calibration range of each particular method. To determine the sensitivity and specificity of different cardiac markers, carefully designed studies are essential; ideally, samples should be collected precisely from ischemic areas of the myocardium, and cases should be carefully selected to ensure they have a well-defined acute ischemic area. This can be accomplished by selecting cases with acute myocardial ischemia confirmed antemortem by clinical observations, ECG, and Troponin T and/or I measurements, and yet who underwent autopsy for some reason. Control cases should include both rapid deaths and those with prolonged agony to allow for fair comparisons. Additionally, the impact of PMI on biomarker stability in cases with significant decomposition warrants further studies, as does the reproducibility of analytical results through repeated analyses to ensure the reliability of internal controls.

## Figures and Tables

**Table 1:** Description of cases, treatment, and analyses in the pilot study. The columns Heart and Serum list the cases in which myocardial homogenates and serum, respectively, were analyzed with the Indiko instrument.

Case	Age	Sex	Cause of Death	Freeze Thaw	HemogloBind	Heart	Serum
P01	77	M	Bronchopneumonia	1	1		
P02	47	M	Lobar pneumonia	1	1		
P03	20	F	Hanging	1	1		
P04	26	M	Intoxication drugs	1	1		
P05	54	M	Hanging	1	1		
P06	18	M	Undetermined	1	1		
P07	55	M	Lung embolism	1			
P08	64	F	Fracture complication	1			
P09	68	F	AMI with rupture	1	1		1
P10	66	M	Bronchopneumonia	1	1		
P11	50	M	Hanging	1	1		
P12	50	M	Hanging	1	1		
P13	59	M	Anoxic brain injury due to AMI		1	1	
P14	45	M	Undetermined		1		
P15	55	M	Rupture of esophageal varices				
P16	60	M	Blood loss due to gastric ulcer		1		
P17	99	M	Coronary arteriosclerosis				
P18	57	M	Hanging		1	1	
P19	61	M	Hanging		1	1	
P20	52	M	Aspiration pneumonia			1	
P21	41	M	Hanging		1	1	
P22	58	M	AMI		1	1	1
P23	78	M	Drowning		1	1	
P24	67	M	Acute pyelonephritis		1	1	1
P25	84	F	Bronchopneumonia		1	1	1
P26	53	M	Traumatic subarachnoid bleeding				
P27	64	M	Hanging		1	1	
P28	60	M	Intoxication drugs and ethanol		1		
P29	56	F	Multiple trauma				
P30	68	M	Intoxication ethanol		1		
P31	76	M	Coronary arteriosclerosis			1	
P32	52	M	Intoxication drugs			1	1
P33	59	F	Intoxication drugs			1	1
P34	92	M	Multiple trauma			1	1
P35	40	M	Dissecting aortic aneurysm			1	1
P36	81	M	Cervical spine injury			1	1
P37	27	M	Intoxication drugs			1	
P38	40	F	Traumatic brain injury			1	1
P39	19	M	Drowning			1	1
P40	83	M	Traumatic brain injury			1	1
P41	83	M	Lobar pneumonia			1	1
P42	49	F	Intoxication carbon monoxide			1	1
P43	40	M	Traumatic brain injury			1	1

**Table 2:** Description of subjects in the main study. Asc LAD and Asc Post = arteriosclerosis in left anterior descending coronary artery and right coronary artery, respectively. BW = body weight. Fibrosis = diffuse or patchy scars in the left ventricular wall. CKD = Chronic Kidney Disease.

Case	Age	Sex	Cause of Death	Group	Height (cm)	BW (kg)	Heart (g)	Asc LAD	Asc Post	Fibrosis	CKD
M01	88	M	Drowning		165	82	492	1	1	1	0
M02	51	M	Hemopericardium		181	92	560	0	0	0	0
M03	20	F	Anoxic brain injury		162	42	205	0	0	0	0
M04	55	M	Bronchopneumonia		171	66	400	1	2	0	0
M05	54	M	Alcoholic ketoacidosis		186	100	394	0	0	0	0
M06	73	M	Lung embolism	Control	179	114	504	0	0	0	1
M07	71	M	Coronary arteriosclerosis	Case	177	57	330	2	2	0	0
M08	75	M	AMI	Case	190	80	505	2	0	0	0
M09	66	F	Hanging	Control	158	48	320	0	0	0	0
M10	38	M	Ashyxia plastic bag	Control	175	69	375	0	0	0	0
M11	64	M	Cardiomegaly	Case	182	138	790	1	0	0	0
M12	73	M	AMI	Case	186	85	540	2	2	2	1
M13	29	F	Hanging	Control	165	49	218	0	0	0	0
M14	53	M	Multiple trauma		186	119	586	0	0	0	2
M15	49	M	TBI		188	128	544	0	0	0	0
M16	55	M	Intoxication opioids		197	136	642	1	1	0	0
M17	38	M	Hanging	Control	181	75	335	0	0	0	0
M18	58	M	Lung cancer		183	95	372	0	0	0	0
M19	52	M	AMI	Case	188	104	490	1	1	0	0
M20	70	M	Blood loss	Control	166	82	450	0	0	0	0
M21	83	M	Fractures with fat embolism		183	62	550	0	1	0	0
M22	49	M	TBI	Control	179	90	470	0	0	0	0
M23	44	M	AMI	Case	185	105	500	1	0	2	0
M24	77	M	Drowning		186	72	556	2	1	1	0
M25	68	M	Intox. drugs and ethanol		185	127	605	0	1	0	0
M26	58	M	Coronary arteriosclerosis	Case	178	85	428	1	1	0	0
M27	64	M	Intoxication drugs		174	73	355	2	1	0	0
M28	52	M	Hanging	Control	180	81	474	0	0	0	0
M29	19	F	Hanging	Control	159	42	185	0	0	0	0
M30	76	F	Heart failure	Case	164	71	530	1	1	1	0
M31	64	M	Possibly SCD		183	74	395	0	0	0	0
M32	65	M	Coronary arteriosclerosis		193	105	582	1	2	1	0
M33	69	M	AMI	Case	183	88	550	2	1	1	1
M34	32	M	Brain hemorrhage	Control	176	73	345	0	0	0	0
M35	36	M	Causa ignota		177	73	350	0	0	0	0
M36	53	M	Coronary arteriosclerosis	Case	184	118	465	1	1	1	0
M37	23	M	Intoxication drugs		183	107	470	0	0	0	0
M38	63	M	Gunshot in the head	Control	174	118	522	0	0	0	0
M39	55	F	Coronary arteriosclerosis		178	98	450	1	0	0	0
M40	29	F	Lung embolism	Control	165	131	414	0	0	0	0
M41	43	M	Cardiomegaly	Case	171	112	596	0	0	2	0
M42	67	M	AMI		167	56	340	2	2	2	1
M43	56	M	Acute pleuritis		170	88	450	0	0	0	0
M44	58	F	Anoxic brain injury		162	84	440	1	2	0	0
M45	57	F	Intox. drugs and ethanol	Control	171	79	405	0	0	0	0
M46	21	M	Intoxication drugs	Control	180	59	310	0	0	0	0
M47	77	M	Cardiomegaly		171	76	520	0	0	0	0
M48	86	F	Bolus death	Control	171	84	482	0	0	0	0
M49	66	F	Alcoholic ketoacidosis		165	47	352	1	1	0	0
M50	72	F	AMI	Case	168	49	500	2	2	0	0
M51	33	M	Hanging	Control	167	77	290	0	0	0	0
M52	39	M	Hanging	Control	170	75	380	0	0	0	0
M53	67	M	Coronary arteriosclerosis	Case	175	93	400	0	0	0	1
M54	18	M	Hanging	Control	185	88	324	0	0	0	0
M55	82	M	TBI		175	68	440	2	1	0	0
M56	49	M	Acute tonsillitis		180	88	530	1	1	0	0
M57	79	M	AMI		183	81	520	2	2	1	0
M58	67	M	Brain hemorrhage	Control	165	49	310	0	0	0	1
M59	53	M	Hanging	Control	181	76	420	0	0	0	0
M60	34	F	Hanging	Control	185	66	440	0	0	0	0
M61	22	M	TBI	Control	165	62	314	0	0	0	0
M62	77	M	Hanging	Control	174	77	430	2	2	0	0
M63	43	M	Burns	Control	174	80	510	0	0	0	0
M64	30	M	Intoxication drugs		193	98	430	0	0	0	0
M65	20	F	Intoxication drugs		181	90	362	0	0	0	0
M66	42	M	Intoxication drugs		200	182	858	0	0	0	0
M67	70	F	Alcoholic ketoacidosis		159	75	514	0	0	0	0
M68	71	F	Diabetic coma		163	45	270	1	1	0	0
M69	34	M	Intoxication drugs	Control	178	80	335	0	0	0	0
M70	73	M	Intoxication drugs		191	158	476	0	0	0	0
M71	68	F	Alcoholic ketoacidosis		160	66	365	0	0	0	0
M72	61	M	Intoxication CO	Control	176	89	395	0	0	0	0
M73	76	M	Coronary arteriosclerosis		173	77	560	2	2	1	1
M74	71	M	Coronary arteriosclerosis		174	70	388	2	2	0	0
M75	76	F	Cardiac arrhythmia		168	70	302	0	0	0	0
M76	63	M	AMI	Case	174	87	465	1	2	0	0
M77	70	F	Intoxication drugs	Control	151	83	425	1	1	0	0
M78	84	F	Intoxication drugs		170	74	516	2	2	1	0
M79	54	M	Intoxication drugs	Control	168	68	320	0	0	0	0
M80	32	M	Ketoacidosis NOS		191	71	342	0	0	0	0
M81	67	F	AMI	Case	159	68	515	2	2	0	0
M82	57	F	Fat embolism		164	43	315	0	0	0	0
M83	65	M	Esophageal bleeding		178	58	295	0	0	0	0
M84	32	M	Intoxication drugs		185	99	460	0	0	0	0
M85	48	M	Hypothermia		167	62	320	0	0	0	0
M86	81	F	Hanging	Control	157	41	342	0	0	0	0
M87	64	M	AMI	Case	177	84	625	2	2	2	2
M88	67	F	Lobar pneumonia—sepsis		170		290	0	0	0	0
M89	65	M	AMI	Case	175	45	574	2	2	1	0
M90	75	F	Spleen injury		157	45	275	0	0	0	0
M91	59	M	Cardiomegaly		189	103	578	0	0	0	0
M92	64	M	Drowning	Control	177	103	380	0	0	0	0
M93	19	F	Intox. drugs + hypothermia		171	49	170	1	1	1	1
M94	82	F	Coronary arteriosclerosis	Case	152	61	695	2	2	1	1
M95	76	F	Cardiomegaly		173	100	635	1	1	0	0
M96	41	M	Cardiomegaly		183	107	550	0	0	0	0
M97	59	M	AMI	Case	185	99	465	1	1	1	0
M98	53	M	Cardiomegaly		179	116	600	0	0	0	0
M99	56	M	Coronary arteriosclerosis		182	122	470	0	0	0	0
M100	56	M	AMI	Case	180	71	482	2	1	0	0
M101	43	M	AMI		179	66	480	0	0	0	0
M102	59	M	Intoxication CO		189	77	476	2	1	1	0
M103	46	F	Multi-organ failure		170	121	498	0	0	0	1
M104	58	M	AMI	Case	186	120	594	1	1	0	0
M105	62	M	Myocardial fibrosis	Case	189	91	516	0	0	2	0
M106	76	M	Coronary arteriosclerosis	Case	175	90	496	2	2	1	0
M107	89	M	Stab wound in heart		175	76	380	0	0	0	0
M108	54	M	AMI	Case	174	75	360	1	1	0	0
M109	22	M	Intoxication drug		182	79	412	0	0	0	0
M110	55	F	AMI	Case	175	68	520	0	0	0	0
M111	76	M	AMI	Case	192	96	740	2	2	1	0
M112	41	M	Lung embolism	Control	183	84	420	0	0	0	0
M113	77	M	AMI	Case	177	71	420	2	2	2	0

Footnote: Coronary arteriosclerosis: grade 0 = absent or soft atheromatosis without luminal narrowing; grade 1 = moderate number and sizes of plaques, occupying <50% of transection area; grade 2 = severe and/or widespread arteriosclerosis with >50% luminal narrowing in at least one of the three main branches. Fibrosis: grade 0 = no fibrosis, or only minute scars; grade 1 = one or more medium-sized scar(s); grade 2 = larger scars or widespread patchy fibrosis, or microscopically significant diffuse myocardial fibrosis. Chronic kidney disease (CKD): grade 0 = no pathologies; grade 1 = macro- and/or microscopic severe nephrosclerosis with reduction of the cortex, macro- and/or microscopic signs of chronic pyelonephritis or other significant kidney pathology indicating reduced renal function. AMI = acute myocardial infarction. TBI = traumatic brain injury. CO = carbon monoxide.

**Table 3:** Concentrations of selected analytes in serum and homogenates. The table shows the levels in both the posterior and anterior myocardial wall in subjects classified as SCD and controls.

Posterior Myocardial Samples									
Analayte	Extraction	SCD Cases				Controls			
		Range	Q1:Q3	Median	N	Range	Q1:Q3	Median	N
ALT	Serum	0.0–984.1	1.9; 19.8	6.5	27	0.1–343.5	5.8; 25.4	16.8	31
AST	Serum	0.0–102.1	2.2; 31.4	8.5	25	0.0–196.8	8.5; 42.4	21.8	26
CK-MB	Serum	0.0–7901	14.4; 85.0	41.7	27	0.3–981.6	36.1; 128.4	66.9	30
LDH	Serum	0.0–3745	17.9; 133.1	72.4	*27*	0.1–1575	86.6; 209.3	133.8	31
Orosomucoid	Serum	0.0–7.4	0.4; 1.1	0.7	27	0.1–8.5	0.1; 1.3	0.9	31
Total Protein	Serum	0.0–686.0	44.0; 79.0	71.0	27	6.0–1176	57.0; 92.0	75.0	31
ALT	dH_2_O	1.0–171.0	82.2; 138.9	109.7	27	42.7–261.0	97; 171.0	132.0	31
AST	dH_2_O	8.0–1299	42.0; 1000	429.3	17	4.3–4632	296; 2697	755.3	13
CK-MB *	dH_2_O	6.7–3664	179.3; 650.6	449.7	27	65.0–120,000	499.3; 2051	1088	31
LDH	dH_2_O	69.7–2198	958.2; 1339.7	1157	27	0.3–2585	1116; 1520	1329	31
Orosomucoid	dH_2_O	0.0–2.8	0.2; 0.5	0.4	21	0.0–3.2	0.2; 1.3	0.6	27
Total Protein	dH_2_O	0.0–40.0	20.0; 20.0	20.0	27	0.0–45.6	20; 20.2	20.0	27
ALT	T-PER	35.8–73.7	72.1; 132.9	110.3	8	38.0–238.0	79.2; 163.0	144.0	30
AST	T-PER	1.0–1054	4.3; 811.0	91.0	27	0.7–943.0	5.1; 733.0	63.2	6
CK-MB	T-PER	4.0–17,925	1487.1; 3740	2715	27	685.0–4906	1537; 3289	2134	30
LDH	T-PER	0.4–1715	726; 1093	938.7	27	227.0–2209	920; 1325	1154	31
Orosomucoid	T-PER	0.0–3.1	0.5; 0.7	0.6	21	0.0–3.2	0.4; 1.4	0.8	29
Total Protein	T-PER	10.0–46.4	20.0; 37.0	20.0	27	0.0–53.0	20.0; 40.0	20.0	31
**Anterior myocardial samples**									
ALT	Serum	0.0–984.0	1.9; 19.8	6.5	27	0.1–343.5	5.8; 25.4	16.8	31
AST	Serum	0.0–102.1	2.2; 31.4	8.5	27	0.0–196.8	8.5; 42.4	21.8	31
CK-MB	Serum	0.0–7901	14.4; 85.0	41.7	27	0.3–981.6	36.1; 128.4	66.9	31
LDH	Serum	0.0–3745	17.9; 133.1	72.4	27	0.1–1575	86.6; 209.3	133.8	31
Orosomucoid	Serum	0.0–7.4	0.4; 1.1	0.7	27	0.1–8.5	0.1; 1.3	0.9	31
Total Protein	Serum	0.0–686.0	44.0; 79.0	71.0	27	6.0–1176	57.0; 92.0	75.0	31
ALT	dH_2_O	1.3–223.7	25.7; 81.3	55.7	21	0.3–211.0	43.3; 139.0	93.7.0	22
AST	dH_2_O	3.3–1390	41.0; 703.3	199.0	19	0.7–1215	65.3; 998.7	215.3	18
CK-MB *	dH_2_O	4.7–1822	42.2; 388.2	68.2	22	22.7–2439	193.3; 1082	559.3	22
LDH	dH_2_O	2.0–2515	982.7; 1977.3	1407	21	0.3–2098	899.3; 1143	1521	22
Orosomucoid	dH_2_O	0.0–0.0	0.0; 0.0	0.0	4	0.0–0.6	0.0; 0.2	0.2	19
Total Protein	dH_2_O	0.0–40.0	20.0; 40.0	20.0	22	0.0–40.0	20.0; 20.0	20.0	25
ALT **	T-PER	39.7–236.0	88.6; 179.3	152.5	22	10.7–189.0	43.3; 112.0	62.0	25
AST	T-PER	0.0–1171	0.3; 855.7	92,3	5	0.3–1217	14.3; 470.0	65.7	17
CK-MB **	T-PER	5.7–17,650	1627; 12,631	3768	22	327.7–4405	851.7; 2455	1262	25
LDH **	T-PER	254.7–1735	919; 1534	1371	22	187.0–1413	436; 784.7	624.7	25
Orosomucoid	T-PER	0.0–0.4	0.2; 0.2	0.2	10	0.0–1.0	0.0; 0.4	0.2	20
Total Protein	T-PER	20.0–60.0	40.0; 40.0	40.0	22	20.0–40.0	20.0; 40.0	40.0	25

Concentration ranges, first (Q1) and third (Q3) quartiles, median and number of observations (N) for the biomarkers measured in postmortem serum and homogenates. * With dH_2_O extraction CK-MB levels were significantly lower in SCD cases than in controls in both posterior and anterior wall samples (*p* = 0.010 and *p* = 0.0061, respectively). ** With T-PER extraction, ALT, CK-MB, and LDH levels were significantly lower in SCD cases than in controls in anterior wall samples (*p* = 0.0029, *p* = 0.026 and *p* = 0.00038, respectively). All comparisons were made with a Mann–Whitney U test with Bonferroni–Holm correction. No significant differences in serum concentrations were seen between cases and controls. With urea pretreatment, too few analytical results were obtained for the cardiac biomarkers to allow for a reliable comparison of groups.

**Table 4:** Degree of coronary arteriosclerosis in the LAD and A. coronary dexter artery, and the levels of select biomarkers in the corresponding myocardial area.

Analyte	Position	ASC Grade 0 + 1				ASC Grade 2			
		Range	Q1:Q3	Median	N	Range	Q1:Q3	Median	N
ALT	LAD vs. anterior wall	4.7–224	59.3; 146	102	28	0.3–265	34.3; 135	87.7	25
AST	LAD vs. anterior wall	8.0–3980	123; 1020	469	20	3.3–4299	36.8; 1242	316	22
CK-MB	LAD vs. anterior wall	14.7–3664	96.3; 3664	425	28	4.7–47,340	60.2; 65	1210	27
LDH	LAD vs. anterior wall	363.0–2499	838; 2499	1278	28	2.0–2515	1111; 1498	1251	26
ALT	A cor. dexter vs. posterior wall	4.7–224	56.6; 141	104	30	0.3–265	37.0; 130	87.6	23
AST	A cor. dexter vs. posterior wall	8.0–3980	74.8; 1000	317	22	3.3–4299	40.3; 1266	513	20
CK-MB	A cor. dexter vs. posterior wall	14.7–3664	99.2; 805	425	30	4.7–47,340	59.7; 702	154	25
LDH	A cor. dexter vs. posterior wall	362.7–2499	1080; 1762	1278	30	2.0–2515	957.0; 1526	1215	24

**Table 5:** Results of proteomics analysis of serum and myocardial homogenates from 10 randomly selected cases, based on mass spectrometry with label-free quantification (LFQ).

Case	Tissue	AST	Myoglobin	TcnT	CK-M-Type	LDH	Orosomucoid	ALT	CK-B-Type
N001	Homogenates	2.8 × 10^9^	2.1 × 10^10^	4.8 × 10^8^	5.1 × 10^9^	6.3 × 10^9^	1.1 × 10^9^	1.6 × 10^8^	8.4 × 10^8^
N003	Homogenates	6.2 × 10^9^	1.3 × 10^10^	2.8 × 10^8^	9.3 × 10^9^	8.5 × 10^9^	4.0 × 10^9^	5.0 × 10^8^	6.0 × 10^7^
N008	Homogenates	1.9 × 10^9^	1.9 × 10^10^	1.3 × 10^9^	4.1 × 10^9^	4.5 × 10^9^	2.2 × 10^9^	1.3 × 10^8^	6.1 × 10^7^
N009	Homogenates	2.8 × 10^9^	2.3 × 10^10^	9.1 × 10^8^	5.7 × 10^9^	5.1 × 10^9^	5.3 × 10^8^	2.6 × 10^8^	3.2 × 10^7^
N011	Homogenates	1.1 × 10^8^	4.2 × 10^10^	1.4 × 10^9^	3.1 × 10^8^	1.0 × 10^8^	1.2 × 10^9^	4.6 × 10^7^	ND
N013	Homogenates	2.7 × 10^9^	4.1 × 10^10^	5.7 × 10^8^	4.1 × 10^9^	1.1 × 10^9^	8.9 × 10^8^	3.7 × 10^8^	6.3 × 10^8^
N040	Homogenates	2.7 × 10^8^	4.9 × 10^10^	1.5 × 10^9^	6.6 × 10^8^	8.0 × 10^8^	2.3 × 10^9^	ND	ND
N044	Homogenates	3.9 × 10^8^	4.9 × 10^10^	1.3 × 10^9^	7.7 × 10^8^	8.5 × 10^8^	4.1 × 10^9^	4.1 × 10^7^	ND
N085	Homogenates	1.4 × 10^7^	4.1 × 10^10^	3.6 × 10^8^	1.7 × 10^8^	5.1 × 10^7^	3.0 × 10^9^	ND	4.2 × 10^7^
N086	Homogenates	1.7 × 10^9^	2.6 × 10^10^	1.2 × 10^8^	5.3 × 10^9^	3.1 × 10^9^	2.0 × 10^9^	1.0 × 10^8^	2.1 × 10^9^
**Mean**		**1.89 × 10^9^**	**3.24 × 10^10^**	**8.24 × 10^8^**	**3.55 × 10^9^**	**3.05 × 10^9^**	**2.13 × 10^9^**	**2.01 × 10^8^**	**5.33 × 10^8^**
N001	Serum	ND	1.4 × 10^9^	ND	ND	ND	5.0 × 10^10^	ND	ND
N003	Serum	ND	1.7 × 10^9^	ND	ND	ND	3.6 × 10^10^	ND	ND
N008	Serum	ND	9.4 × 10^7^	ND	ND	ND	5.4 × 10^10^	ND	ND
N009	Serum	ND	2.8 × 10^8^	ND	ND	4.0 × 10^7^	3.1 × 10^10^	ND	ND
N011	Serum	ND	2.9 × 10^8^	ND	ND	ND	3.4 × 10^10^	ND	ND
N013	Serum	1.3 × 10^7^	9.4 × 10^8^	ND	1.2 × 10^8^	2.4 × 10^7^	2.8 × 10^10^	ND	ND
N040	Serum	ND	7.6 × 10^8^	ND	4.8 × 10^7^	1.5 × 10^7^	2.6 × 10^10^	ND	ND
N044	Serum	ND	3.4 × 10^9^	ND	8.9 × 10^7^	ND	2.5 × 10^10^	ND	ND
N085	Serum	ND	4.7 × 10^9^	ND	9.0 × 10^7^	ND	2.5 × 10^10^	ND	ND
N086	Serum	8.6 × 10^7^	1.4 × 10^9^	ND	5.3 × 10^7^	4.1 × 10^7^	2.0 × 10^10^	ND	ND
**Mean**	**Serum**	**4.93 × 10^7^**	**1.49 × 10^9^**	**ND**	**7.98 × 10^7^**	**2.939 × 10^7^**	**3.31 × 10^10^**	**ND**	ND

## Data Availability

The data presented in this study are available on request from the corresponding author due to access restrictions by the owner of the original data, the Swedish National Board of Forensic Medicine. Although the cases are coded, there is linked information to each case, such as date of death and circumstances, which may disclose the identity of the subject, so before disclosure of data upon request, a filtering of certain information in the research data master file will be necessary.

## Abbreviations

The following abbreviations are used in this manuscript:

SCD	Sudden Cardiac Death
ALT	Alanine aminotransferase
AST	Aspartate aminotransferase
CK-MB	Creatine Kinase-MB
LDH	Lactate Dehydrogenase
NT-proBNP	N-terminal pro-B-type Natriuretic Peptide
PMI	Post-Mortem Interval
CRP	C-Reactive Protein
AMI	Acute Myocardial Infarction
MR	Magnetic Resonance
tcPMI	Temperature Corrected Post-Mortem Interval
PBS	Phosphate-Buffered Saline
SDS	Sodium Dodecyl Sulfate
NADH	Nicotinamide Adenine Dinucleotide
NADPH	Nicotinamide Adenine Dinucleotide Phosphate
ECLIA	Electrochemiluminescence Immunoassay
cTnT	Cardiac Troponin T
ECG	Electrocardiogram
CT	Computed Tomography
